# Co-infections and Pathogenesis of KSHV-Associated Malignancies

**DOI:** 10.3389/fmicb.2016.00151

**Published:** 2016-02-15

**Authors:** Suhani Thakker, Subhash C. Verma

**Affiliations:** University of Nevada School of MedicineReno, NV, USA

**Keywords:** KSHV, herpesviruses, polymicrobial infections

## Abstract

Kaposi’s sarcoma-associated herpesvirus (KSHV), also known as human herpes virus 8 (HHV-8) is one of the several carcinogenic viruses that infect humans. KSHV infection has been implicated in the development of Kaposi’s sarcoma (KS), primary effusion lymphoma, and multicentric Castleman’s Disease. While KSHV infection is necessary for the development of KSHV associated malignancies, it is not sufficient to induce tumorigenesis. Evidently, other co-factors are essential for the progression of KSHV induced malignancies. One of the most important co-factors, necessary for the progression of KSHV induced tumors, is immune suppression that frequently arises during co-infection with HIV and also by other immune suppressants. In this mini-review, we discuss the roles of co-infection with HIV and other pathogens on KSHV infection and pathogenesis.

## Introduction

Kaposi’s sarcoma-associated herpes virus (KSHV), the latest member of the human herpes virus family has been classified as a direct carcinogen by the International Agency for Research on Cancer (Ignatovich et al., 2002) because of its ability to induce sarcomas and lymphomas (IARC Working Group on the Evaluation of Carcinogenic Risks to Humans, 2012; Chen et al., 2014). Based on consistent detection of KSHV genome in Kaposi’s sarcoma (KS) and two lymphoproliferative disorders named Primary effusion lymphoma (PEL) and plasmablastic variant of multicentric Castleman’s Disease (MCD), KSHV infection has been linked with these malignancies (Chang et al., 1994; Cesarman et al., 1995; Soulier et al., 1995; Moore and Chang, 2010). Upon infection, KSHV establishes long-term persistent infection in humans where it manipulates multiple host signaling pathways, modulates expression of cellular genes, and perturbs cellular homeostasis in order to thrive in the infected cells (Jarviluoma and Ojala, 2006; Moore and Chang, 2010; Schafer et al., 2015). Continued cellular homeostasis disturbances associated with KSHV infection can potentially lead to the development of cancers, however, not all the KSHV infected humans develop these malignancies, emphasizing the importance of co-factors in determining the development, and extent of severities of KSHV associated diseases. Since humans serve as natural hosts to an enormously diverse species of microbes, the complex interplay of co-infecting microbes can influence the outcomes of pathologies associated with KSHV infection. The co-infecting microbes may interact in multifaceted ways such as direct molecular interactions and indirect modulation of the microenvironment including immune modulation and cytokine deregulation. Co-infection with other pathogens is likely to be an important determinant of disease progression associated with KSHV infection but unfortunately, knowledge regarding the impact of co-infecting pathogens on KSHV associated pathologies are not very clear. Here, we summarize the current information regarding the impact of several co-pathogenic infections on KSHV infection and the associated pathologies.

## An Overview of KSHV Biology

Humans are the natural host of the oncogenic herpesvirus KSHV. In immune-competent individuals, KSHV establishes asymptomatic life-long latency following acute infection, however, it can cause cancers in the infected individuals with compromised immunity. The virus has a diverse range of *in vivo* and *in vitro* cell tropism but CD19+ B cells appear to be the primary target for long-term viral latency (Ambroziak et al., 1995; Lukac and Yuan, 2007; Veettil et al., 2014). Approximately 165 Kb of double stranded DNA genome of KSHV encodes for about 90 open reading frames, 12 precursor micro RNAs (pre-miRNAs) that are spliced into at least 25 mature miRNAs, and a number of non-coding and antisense RNAs (Russo et al., 1996; Ganem, 2007; Longnecker and Neipel, 2007; Martin, 2007; Cai et al., 2010; Arias et al., 2014; Bhutani et al., 2015; Hu et al., 2015). Based on the expression profiles of the viral genes, the life cycle of KSHV is divided into two distinct phases, latent and lytic (Miller et al., 1997; Parravicini et al., 2000; Dourmishev et al., 2003; Edelman, 2005; Guito and Lukac, 2015). Latency is a non-productive phase characterized by the restricted gene expression that aids the virus in avoiding host immune recognition while allowing for long-term viral persistence (Guito and Lukac, 2015; Hughes et al., 2015). Amongst the latently expressed genes, latency associated nuclear antigen (LANA/LANA-1/ORF73) is the most abundantly expressed protein consistently detected in all latently infected tumors. Expression of LANA is absolutely essential for the maintenance of KSHV latency because of its pleiotropic roles including replication and maintenance of the viral genome, host cell survival, proliferation, and immune evasion (reviewed in Giffin and Damania, 2014; Uppal et al., 2014). Lytic phase is characterized by the expression of a highly ordered cascade of viral genes that ensures efficient replication of the viral DNA and its packaging into the new virions. Lytic replication is essential not only for transmission and dissemination the virus, but also is considered to be a critical step in the development of KSHV induced cancers (Lukac and Yuan, 2007; Giffin and Damania, 2014; Hughes et al., 2015; Purushothaman et al., 2015). The switch from latent to lytic infection, is a tightly regulated process initiated by the expression of KSHV ORF50/RTA, the lytic switch protein considered both necessary, and sufficient to drive lytic replication (Ye et al., 2011; Purushothaman et al., 2015). A large portion of the KSHV genome is kept silenced during latency through multiple epigenetic modifications including histone deacetylation and repressive histone methylations. However, during lytic replication, the levels of histone acetylation increases and repressive histone methylation marks are replaced with activating histone methylation marks on the viral genome, allowing for the expression of lytic genes (Pantry and Medveczky, 2009; Toth et al., 2010; Hu et al., 2014; Yu et al., 2014). Some of the well-known *in vivo* factors that activate lytic replication of KSHV include cellular stresses, hypoxia, inflammation, co-pathogenic infections, apoptosis and the immune suppression state of the infected host (reviewed in Uppal et al., 2014; Purushothaman et al., 2015). Amongst these, immune status of the infected host is one of the key factors that controls viral reactivation; a healthy immune system controls KSHV lytic reactivation and enforces latency (Lukac and Yuan, 2007). Appropriate regulation of latent and lytic gene expression is extremely critical for viral persistence and spread, disturbances in the regulation of these mechanisms can lead to a development of cancers. Co-pathogenic infections have a potential to perturb these regulatory mechanisms in a variety of ways and thus influences the outcomes of the pathologies associated with KSHV infection. Effects of several co-infecting pathogens on KSHV infection and associated pathologies are summarized here.

## KSHV Induced Pathologies

Kaposi’s sarcoma-associated herpesvirus infection is linked to several malignancies in humans (Kalt et al., 2009). KSHV infection of endothelial cells lays a foundation for the development of Kaposi’s Sarcoma (KS), a highly vascularised tumor of endothelial origin (Ganem, 2006), and the infection of B cells can cause a rare but aggressive B cell tumor, PEL (Cesarman et al., 1995). A variant of MCD is another disease associated with KSHV infection (Chang et al., 1994; Soulier et al., 1995). More recently, KSHV-inflammatory cytokine syndrome (KICS) has been described as a new inflammatory disorder associated with KSHV infection (Uldrick et al., 2010).

Kaposi’s sarcoma is a slow-growing tumor of endothelial origin thought to be dependent on viral replication, immune modulation and production of multiple inflammatory cytokines by the infected endothelial as well as the immune cells (Knowlton et al., 2014). Interestingly, KS development has been skewed toward male population. Four histologically indistinguishable subtypes of KS have been described, (i) classic KS, a rarely aggressive form predominantly observed in elderly men of Mediterranean and Middle Eastern ancestry, (ii) African-endemic KS, associated with significant morbidity and mortality, (iii) iatrogenic KS, frequently found in patients with pharmacological immune suppression, and (iv) HIV-1 co-infection associated endemic KS (AIDS-KS), commonly observed in AIDS patients (reviewed in Schulz and Cesarman, 2015). Of these, AIDS-KS has the most aggressive clinical manifestations of all KS subtypes and has become one of the most common cancers in many Sub-Saharan countries where both, HIV and KSHV are endemic (Giffin and Damania, 2014).

Traditionally KS was considered an AIDS-defining malignancy because the patients who were at highest risk of developing KS were HIV-1 co-infected patients with high HIV-1 viral load and very low peripheral CD4+ T cell counts (<200 cells/mm^3^), a count that defines patient’s progression into AIDS. Following the introduction of combination antiretroviral therapies (cART), which are very effective in controlling HIV-1 replication and in restoring CD4+ T cell counts in HIV-1 co-infected patients, incidences of KS in HIV-1 infected populations have substantially declined but not quite eradicated (Labo et al., 2015). Enigmatically, despite of the steep decrease in overall incidences of KS in post-cART era, there is a steady increase in proportions of KS patients diagnosed at CD4+ T cell counts in excess of 300 cells/mm^3^, a count generally not associated with complications arising from opportunistic infections (Maurer et al., 2007; Krown et al., 2008; Mani et al., 2009; Daly et al., 2014; Labo et al., 2015). This may be due the fact that highly effective cART has significantly extended the life expectancy of HIV-1 infected patients; the observed incidences of KS at higher CD4+ T cell counts may be senescence related entity resembling the classic KS. Another possibility is that HIV-1 co-infection may have additional influence on the development of KS independent of its effects on suppression of CD4+ T cell immunity; several such potential mechanisms are discussed later in this review. Regardless of the underlying mechanism, observed and expected increase in the cases of KS development at higher CD4+ T cell counts warrants continued research in the field of KSHV in order to reduce the risk of developing such cancer.

Primary effusion lymphoma is a rare but rapidly progressing B cell tumor (Simonelli et al., 2003; Ota et al., 2014). PEL typically presents as malignant effusions without solid tumor mass within the pericardial, pleural, and peritoneal body cavities of infected patients but the incidences of extra cavity PEL representation as mass lesions have also been reported (Deloose et al., 2005; Pan et al., 2012; Crane et al., 2014). It is an aggressive lymphoma with a poor prognosis and average time of only 6 months from the diagnosis. PEL usually arises in the context of HIV co-infection or other immune suppressed states such as in recipients of solid organ on immune suppressants (Kim et al., 2014). EBV co-infection appears to play an active role in the progression of PEL based on the observation that majority of PEL patients are also co-infected with EBV (Boulanger et al., 2005).

Plasmablastic version of MCD is a polyclonal tumor where lytic replication of KSHV in lymph node plasmablasts promotes excessive production of pro-inflammatory cytokines. KSHV-MCD is characterized by hypercytokinemia, lymphadenopathy, splenomegaly, and displays a variety of inflammatory symptoms which can be life threatening in the absence of proper treatment (Polizzotto et al., 2012; Fajgenbaum et al., 2014). Progression of KSHV-MCD is much more aggressive in KSHV/HIV co-infected patients compared to HIV negative patients suggesting an active role of HIV in accelerating the progression of KSHV-MCD (Suda et al., 2001; Pinzone et al., 2015). KSHV-MCD is associated with elevated KSHV viral loads in their peripheral blood and is accompanied by excessive production of viral interleukin 6 (vIL6), human interleukin 6 (hIL6), and interleukin 10 (IL10) and their cooperative effect play an important role in MCD pathogenesis (Oksenhendler et al., 2000; Polizzotto et al., 2012). Interestingly, these cytokines are potent pro-inflammatory cytokines that can deregulate the control mechanisms of not only KSHV but also the co-infecting pathogens to aggravate pathologies associated with the co-infections.

KSHV Induced Cytokine Syndrome (KICS) is a similar inflammatory condition arising as a result of severe systemic lytic reactivation of KSHV. It is associated with high systemic viral load and excessive cytokine production, notably, elevated levels of interleukin 6 (IL-6), and interleukin 10 (IL-10) (Uldrick et al., 2010; Ballon et al., 2015). These features of KICS are comparable to the KSHV-MCD without the characteristic lymphadenopathy (Ballon et al., 2015).

Kaposi’s sarcoma-associated herpesvirus oncogenicity can be ascribed to its astounding capacity to encode several genes and miRNAs that target cell survival, apoptosis, and tumor suppressor pathways (reviewed in Ganem, 2007; Cai et al., 2010; Schulz and Cesarman, 2015). KSHV’s cell transforming capability has been demonstrated by its ability to transform several primary and immortalized cells (Flore et al., 1998; An et al., 2006; Mutlu et al., 2007; Jones et al., 2012). Despite of demonstrated oncogenic activity of KSHV in a multitude of experimental systems, the fact that only a subset of KSHV infected people develop tumors, strongly suggests that additional factors are required for causing cancers. Co-pathogenic infections are emerging as important co-factors in inducing KSHV related oncogenesis, however, the underlying mechanisms of pathogenic co-operations on KSHV associated malignancies are still not well defined. Based on current knowledge, the process of polymicrobial synergy contributing to the disease progression can be broadly classified into three major categories, (i) deregulation of host immunity associated with the infecting co-pathogens, (ii) direct interactions between co-infecting microbial products, and (iii) indirect synergistic mechanisms involving the modulation of tumor microenvironment through secreted products. Enormously complex tangles of these mechanisms contribute to a progression of KSHV induced cancers during co-pathogenic infections; impact of a few of the well-studied co-infecting pathogens on KSHV associated oncogenesis is detailed below.

## HIV-1 Co-Infection

HIV-1 is one of the most important co-pathogen known to aggravate KSHV induced pathologies (Bhutani et al., 2015). Numerous studies have demonstrated that KSHV infected individuals who are also HIV-1 seropositive, are at substantially higher risk of developing KS cancers compared to HIV-sero-negative people (Newton et al., 2003; Bonnet et al., 2004; Grulich et al., 2007; Rohner et al., 2015). Furthermore, AIDS related KS is clinically most aggressive form and is also more difficult to treat compared to the other forms of KS. Incidences of MCD and PEL tumors are also higher in HIV co-infected patients compared to KSHV infected HIV-1 negative patients (Engels et al., 2008; Powles et al., 2009; Krause et al., 2014; Okada et al., 2014). One of the main effects of uncontrolled HIV infection is severe deregulation of both adaptive and innate host immunities, a very important co-factor contributing to the progression of KSHV pathologies. HIV-1 primarily establishes latency in resting memory CD4+ T cells and in the cells of monocytic/macrophage lineage of which CD4+ T cells are highly susceptible to HIV-1 induced cytopathic effects (Abbas et al., 2015). Progressive loss of CD4+ T cells, thought to stem from chronic immune activation is a major hallmark of HIV-1 co-infection (Sastry et al., 1996; Paiardini and Muller-Trutwin, 2013; Kumar et al., 2015; Younas et al., 2016). Apparently, CD4+ T cell immunity plays a crucial role in controlling KSHV lytic replication and consequently is important for limiting pathogenesis associated with KSHV.

HIV-1 encoded Tat (transcriptional transactivator) is a small cationic peptide that can penetrate a variety of cells (Rusnati and Presta, 2002). It has multiple regulatory roles in HIV-1 replication and host immune deregulation (Fiume et al., 2015). Several effects of HIV-Tat on KSHV infection have been noted. HIV-Tat can enhance KSHV infectivity, promote angiogenesis, and can interact with KSHV proteins to modulate multiple signaling pathways (Aoki and Tosato, 2007). Tat is frequently detected in KS spindle cells where it might promote the growth of spindle cells. Another demonstrated effect of Tat is inducing apoptosis of CD4+ T cells (Li et al., 1995; Sastry et al., 1996). Increased risk of developing KS and PEL associated with low CD4+ T cell count indicates that immune suppression associated with HIV co-infection plays essential roles in KSHV induced pathologies (Mbulaiteye et al., 2003; Rodriguez Salazar et al., 2004; Biggar et al., 2007; Engels et al., 2008). Interestingly, presentation of MCD seems to be independent of CD4+ T cell counts, suggesting that additional factors are necessary for the progression of this disease in HIV-1 co-infected patients (Mylona et al., 2008). Considering the importance of weakened host immunity in inducing KSHV associated pathologies, reconstitution of host immune system after HAART/cART treatment in HIV infected individuals is expected to improve clinical outcomes of KSHV pathologies, but occasionally it can paradoxically worsen manifestation of KS in a condition called KSHV associated immune reconstitution inflammatory response (IRIS-KS; Shelburne et al., 2002; Letang et al., 2013). Paradoxical worsening of PEL and MCD with IRIS has not been yet reported. Suppressed immunity associated with HIV co-infection also renders the host susceptible to a multitude of opportunistic infections, many of which can reactivate KSHV and negatively impact clinical outcomes of KSHV associated malignancies.

While immune suppression associated with HIV infection appears to be one of the most important aspects of HIV’s capacity to induce tumors, reports suggest that it is probably not the only mechanism that favors KSHV driven oncogenesis (Gallo, 1998). For example, studies aimed at investigating the distribution of KS cases in patients with HIV-1 and HIV-2 infections in Gambia, West Africa, where KSHV infection is endemic, suggested that KS developed almost exclusively in HIV-1 co-infected patients despite of similar levels of immune suppression and KSHV sero-prevalence in both HIV-1 and HIV-2 infected groups (Ariyoshi et al., 1998; Zeng et al., 2007). In another study, the risk of developing KS in AIDS patients was found to be several fold higher compared to the pharmacologically immune suppressed patients with organ transplant (Mercader et al., 2000).

Chronic inflammation associated with HIV-1 infection may also contribute to HIV-1’s ability to induce tumors in KSHV infected patients (Fiume et al., 2015). In AIDS-KS, there is a notable increase in inflammatory cytokines such as tumor necrosis factor a (TNF-a), interferon γ (IFN-γ, interleukins 1 and 6 (IL-1 and IL-6). HIV-1 Tat can penetrate a variety of target cells to induce the expression of multiple cytokines and growth factors, some of which can reactivate KSHV lytic replication cycle (Gallo, 1998; Zeng et al., 2007; Debaisieux et al., 2012). Release of these inflammatory cytokines and soluble factors foster a microenvironment that is conducive for KS tumors by promoting angiogenesis and cellular proliferation, or by deregulating apoptosis (Gallo, 1998; Douglas et al., 2010). For example, IL-6 and IL-10 induced by Tat in lymphoid cells may serve as autocrine growth factors for MCD and PEL, respectively (Aoki and Tosato, 2007; Pinzone et al., 2015). Angiogenesis is an essential component of KS tumorigenesis. HIV-1 Tat is postulated to play important roles in initiation and progression of KS in AIDS patients by promoting angiogenesis through induction of various cytokines. HIV-1 Tat itself serves as a potent angiogenic factor that can co-operate with vascular endothelial growth factor (VEGF) for the activation of KS precursor spindle and endothelial cells (Albini et al., 1996). Tat also synergizes with KSHV vIL6 to promote angiogenesis and tumorigenesis through the regulation of PI3K/PTEN/GSK-3β signaling pathway (Zhou et al., 2013). Moreover, Tat potentiates tumorigenesis through its cooperation with KSHV oncoproteins, vGPCR and Kaposin A (Guo et al., 2004; Chen et al., 2009). Tat has been shown to promote growth of endothelial cells derived from KS lesions of AIDS-KS patients (Ensoli et al., 1990). Very recently, a complex synergy between HIV-1 Tat and KSHV lytic oncogene K1 has been shown to promote angiogenesis through a mechanism involving an activation of NF-κB signaling pathway through the inhibition of IκBα resulting from synergistic induction of cellular miR8911-5p by Tat and K1 (Yao et al., 2015). HIV-1 Nef is another secreted protein that can be taken-up by a variety of cell types. Nef has been shown to cooperate with KSHV vIL6 in order to induce cell proliferation and tumorigenesis by inducing AKT signaling (Xue et al., 2014). As mentioned earlier, lytic reactivation of KSHV from latency is thought to be a critical step in promoting KSHV associated pathologies. HIV-1 infection can reactivate latent KSHV genomes either directly, or through enhanced production of multiple cytokines (Greene et al., 2007). In a co-culture study of HIV-1 infected T cells (CEM) and latently KSHV infected B cells (BCBL-1), several cytokines including oncostatin M (OSM), hepatocyte growth factor/scatter factor (HGF/SF), and IFN-γ were found to be induced in response to HIV-1 infection in CEM or BCBL-1 cells. Furthermore, these cytokines were able to trigger KSHV lytic replication from latent KSHV genomes (Mercader et al., 2000). HIV-1 infection has also been shown to directly reactivate KSHV in experimental settings. Multiple studies have demonstrated that latently KSHV infected PEL cell line, BC-3 can be infected by HIV-1 and these dually infected cells can support productive replication of HIV-1. HIV-1 co-infection of BC-3 can directly reactivate latent KSHV genomes through potent induction of KSHV lytic master switch protein, RTA (Varthakavi et al., 1999, 2002; Merat et al., 2002). The effects of Tat on reactivation of latent KSHV genomes are partially attributable to Tat’s ability to induce inflammatory cytokines and synergize with KSHV proteins. In addition to directly or indirectly activating KSHV lytic replication, HIV-1 infection can also enhance infectivity of KSHV virions in endothelial and other cells through the expression of Tat, most likely by concentrating KSHV virions on the surface of the cell (Aoki and Tosato, 2004). Reciprocally, KSHV infection can also induce robust replication of HIV-1 *in vivo* and *in vitro* (Caselli et al., 2001, 2003, 2005; Mercader et al., 2001). Interestingly, KSHV is capable of infecting human tonsillar CD4+ T cells *ex-vivo*, the primary targets of HIV-1 infection, although KSHV infection in these cells seems to be abortive (Myoung and Ganem, 2011). This finding is important because it highlights KSHV’s potential to influence HIV-1 biology in HIV-1’s primary target CD4+ T cells (Karijolich et al., 2014). Several KSHV proteins including KSHV RTA/ORF50, LANA/ORF73, and KIE-2/ORF45 have been shown to modulate the activity of the long terminal repeat region (LTR) of HIV-1 through their cooperative actions with HIV-1 Tat (Huang et al., 2001; Hyun et al., 2001; Caselli et al., 2003; Karijolich et al., 2014). In addition to boosting-up HIV-1 replication, these interactions enhance the susceptibility of cells to HIV-1 infection. For example, KSHV ORF50/RTA stimulates HIV replication in HIV permissive T and B cells, but it also induces transient permissiveness of HIV-1 infection in HIV-1 non-susceptible glial cells (Caselli et al., 2003). When HIV-1 and KSHV genomes co-exist in the same cellular environment, both the viruses increase each other’s gene expression through active bidirectional talks (Huang et al., 2001). KSHV ORF45 has been identified as a part of amplification loop for activating HIV-1 LTR in cooperation with HIV-1 Tat and Vpr. KSHV-ORF45 stimulates the expression of Tat and Vpr and these proteins return the favor by inducing the expression of KSHV protein ORF45. KSHV infection thus may serve as an important co-factor for HIV-1 progression. While tumor cells naturally co-infected with HIV-1 and KSHV have not been identified yet, these findings support a possibility of existence of HIV-1 and KSHV dually infected reservoirs in natural settings. Both the viruses can infect cells of monocytic/macrophage, B-cell, and T-cell lineages (Merat et al., 2002; Myoung and Ganem, 2011). Interestingly, a direct molecular interaction between HIV-1 and KSHV components has also been observed in experimental systems. The carboxy terminal domain of LANA physically associates with Tat protein *in vitro* to induce a robust transactivation of LTR by targeting the core enhancer element of LTR (Hyun et al., 2001; Karijolich et al., 2014). Existence of direct molecular interaction between these two viruses suggests that they may have evolved to interact with each other in nature and co-existence of both the viruses in the same cellular compartments that are yet to be identified is likely. Collectively, all these findings provide a substantial evidence for existence of indirect as well as direct molecular interactions between KSHV and HIV-1 that can stimulate carcinogenesis in the host co-infected with both viruses. Amongst these interactions, HIV-1 Tat seems to exert multiple immunomodulatory, angiogenic, cell-signal network perturbing effects, which makes it an attractive therapeutic target for HIV-1 and KSHV co-infected individuals. Active bidirectional talk between these two viruses can partially explain exorbitantly high incidences of KS in HIV-1 infected population and increased opportunistic infections observed in the patients dually infected with both of these viruses. Detailed understanding of the mechanisms of interactions between HIV-1 and KSHV may help in identifying undesirable synergistic interactions between the two viruses that can be pharmacologically uncoupled to limit the damage associated with infections of both of these viruses. This is particularly relevant in the resource-limited settings of Sub-Saharan countries where KSHV and HIV-1 infections are endemic and KSHV associated cancers still remain a public health burden because of limited access to cART (Orem et al., 2004; Rohner et al., 2014).

## Epstein-Barr Virus (EBV, HHV-4) Co-Infection

Epstein-barr virus belongs to the same human γ-herpesvirus family as KSHV. Like KSHV, EBV is also an oncogenic lymphotropic virus associated with infectious mononucleosis and several cancers including an epithelial tumor nasopharyngeal carcinoma, a B-cell neoplasm, Burkitt’s lymphoma, and gastric carcinoma (Kempkes and Robertson, 2015). Similar to KSHV associated malignancies, risk of cancers associated with EBV infection is higher in immune compromised hosts, including HIV co-infected individuals. *In vivo* cell tropism for EBV and KSHV overlap for B-cells but differ for epithelial (EBV) and endothelial cells (KSHV). Direct molecular interactions between KSHV and EBV can occur during chronic viral infections of B-lymphocytes, a natural latency target for both of these oncogenic viruses. While KSHV infection is a pre-requisite for PEL development and EBV infection is not, it is interesting to note that nearly 70% of PEL cell lines are co-infected with EBV. Both of these viruses may make the cellular microenvironment more hospitable for each other and direct or indirect interactions between them may influence pathogenesis of PELs (Drexler et al., 1998; Trivedi et al., 2004; Spadavecchia et al., 2010). Indeed, a couple of studies have demonstrated that EBV co-infected PEL cell lines are more tumorigenic compared to the EBV negative PEL cell lines (Boshoff et al., 1998; Trivedi et al., 2004).

Several studies have investigated molecular interactions between two viruses and the results have provided fascinating insights into the direct molecular interactions between two viruses. Apparently, these two viruses can suppress lytic replication of each other in dually infected cells (Krithivas et al., 2000; Xu et al., 2007; Jiang et al., 2008; Spadavecchia et al., 2010). Like KSHV, EBV also primarily establishes latency upon infection in B lymphocytes and periodically reactivates to lytic replication cycle for production and dissemination of the virus (Murata, 2014). An immediate-early protein of EBV, Zta (EBV-Z/BZLF1/ZEBRA/EB1) initiates the lytic replication cascade of EBV (Wang et al., 2009). In KSHV and EBV dually infected cells, this EBV protein physically interacts with KSHV lytic cycle initiator protein, RTA/ORF50 (K-RTA). The interaction occurs through leucine heptapeptide repeat (LR) region of K-RTA and leucine zipper region of EBV-Z resulting into an inhibition of both the molecules and consequent inhibition of respective lytic replication cycles. KSHV-RTA or EBV-ZTA, whichever is predominantly expressed, can effectively suppress the other virus’s lytic replication and in the case of PEL cell lines, KSHV appears to be the dominant genome (Jiang et al., 2008; Ueda et al., 2011). Another distinct mechanism leading to a suppression of KSHV lytic replication by EBV involves an EBV latent membrane protein 1 (LMP1), which is a major EBV oncoprotein essential for the establishment of EBV latency and for the oncogenic processes driving B-cell transformations (Jiang et al., 2008; Kempkes and Robertson, 2015). KSHV-RTA induces the expression of EBV-LMP1 in latently EBV infected cells and LMP-1 in turn suppresses KSHV lytic replication by downregulating KSHV-RTA expression. While EBV-ZTA suppresses the lytic reactivation of only KSHV, expression of EBV-LMP1 suppresses the lytic replication of both, EBV and KSHV. Furthermore, KSHV can reinforce EBV latency by transactivating the EBV latency promoters through K-RTA through RBP-Jk, the cellular DNA-binding component of the Notch signal transduction pathway (Spadavecchia et al., 2010). An indirect mechanism of latent/lytic modulation of these two viruses during co-infections has also been noted. A recent study showed that activation of signal transducer and activator of transcription 3 (STAT-3), a potent cellular transcription factor that is up-regulated independently during both KSHV and EBV infections, can suppress lytic replication of KSHV through the suppression of KSHV-RTA promoter (Lui et al., 2009; King et al., 2015). This seems to be dependent on the activation of the cellular transcriptional co-repressor Krüppel-associated box domain-associated protein 1 (KAP1/TRIM28; King et al., 2015). Since several of the latent viral proteins of both these viruses are tumorigenic and because long-term latency offers prolonged periods of synergistic actions of these proteins including modulation of cell signaling and induction of inflammatory cytokines; co-infection with both the viruses may enhance the tumorigenic potential of the co-infected cells.

Both of these oncogenic viruses may have evolved various mechanisms for mutual suppression of lytic reactivation in order to compete for limited resources available during unfavorable cellular conditions, however, long-term latency resulting from the co-infection of both of these viruses may also offer mutual survival advantages by helping them hide from the radar of the host’s immune surveillance. For example, several of the EBV encoded microRNAs (miRNAs) target crucial cellular pathways including apoptosis, cell-cycle control and immune-modulation pathways whose deregulation is important for long-term viral persistence of both KSHV and EBV (Gottwein et al., 2011; Riley et al., 2012; Haecker and Renne, 2014). Interestingly, there is a significant functional overlap between the miRNAs of KSHV and EBV in repressing the same cellular pathways despite of structural disparities between the miRNAs of these viruses. This functional synergy underscores the importance of co-infections in modulating host microenvironment to favor the viral persistence.

In addition to promoting EBV latency, KSHV infection can also enhance the infectivity of EBV indicating that EBV co-infection may confer survival advantages to KSHV. In KSHV infected B-lymphocytes, K-RTA has been shown to increase the infectivity of EBV through the upregulation of CD21 expression, a receptor for EBV entry (Chang et al., 2005).

## Human Cytomegalovirus (HCMV, HHV-5) Co-Infection

Human cytomegalovirus is an archetypical member of β-herpesvirus subfamily. HCMV infection is highly prevalent worldwide but has variable seropositivity rates, and is endemic in developing countries, especially in sub-Saharan Africa where KSHV infection is also endemic (Adland et al., 2015). Following primary infection, HCMV can establish latent infection in hematopoietic progenitor cells (HPCs) or in monocytes (Tarrant-Elorza et al., 2014). Primary infection with HCMV is largely asymptomatic in healthy individuals, but its reactivation could cause significant morbidity and mortality in immune compromised patients; notably, these patients are also at higher risk for developing KSHV associated malignancies. HCMV has a very diverse cell tropism including hematopoietic cells, parenchymal cells, epithelial cells, endothelial cells, fibroblasts and smooth muscle cells (Sinzger et al., 2008). Of these, epithelial cells, endothelial cells, fibroblasts and smooth muscle cells are the predominant targets for bursts of lytic replication (Sinzger et al., 2008; Tarrant-Elorza et al., 2014). Notably, monocytes support long-term latency for both of these viruses. Since HCMV cell tropism coincides significantly with KSHV cell tropism, *in vivo* interactions between these two viruses, although not discovered yet, are likely to exist. Presence of HCMV has been detected in KS lesions and experimental evidences suggest that HCMV co-infection can induce KSHV lytic replication. Co-infection of HCMV in experimental systems is known to activate KSHV lytic replication cycle in a variety of cells including endothelial cells, fibroblasts, and keratinocytes (Vieira et al., 2001; Lu et al., 2005; Wells et al., 2009). The mechanism of KSHV reactivation by HCMV involves an activation of KSHV-RTA by UL112-113 region of HCMV that encodes four nuclear phosphoproteins via alternative splicing with essential roles in HCMV DNA replication (Park et al., 2006). Although it is unclear whether these HCMV proteins interacts with the RTA promoter directly (Wells et al., 2009). HCMV infection thus may influence KSHV associated pathogenesis in the host co-infected with both the viruses. Mutual interactions between KSHV and HCMV, although not well studied, hold a high significance in the context of immune suppression, which is a strong co-factor for the reactivation of both the viruses and the progression of associated pathologies. These interactions are especially more relevant in sub-Saharan Africa where HIV, KSHV, and HCMV all are endemic.

## HHV-6 Co-Infection

Human herpesvirus-6 is another non-oncogenic herpesvirus belonging to the β-herpesvirus subfamily. The virus is classified into two variants, HHV-6A and HHV-6B (Ablashi et al., 2014). Primary infection of HHV-6B is associated with exanthem subitum (also known as roseola infantum) and other febrile illnesses, while pathogenesis of HHV-6A is not well defined (Yamanishi et al., 1988; Mori et al., 2015). HHV-6 infection is widespread. Primary infection of the virus is thought to occur before the age of 3 years in most children, and nearly 95% of adult population is estimated to be infected with this virus (Zerr et al., 2005). Following primary infection, HHV-6 establishes a life-long latency, which can reactivate to lytic replication during the phases of immune suppression. Viral reactivation often has severe manifestations in immune-compromised patients; HHV-6 lytic reactivation has been implicated in hepatitis, pneumonitis, reactivation of cytomegalovirus (CMV), fever and rash, myelosuppression, and encephalitis (Zerr et al., 2005). The virus has a broad *in vivo* and *in vitro* cell tropism; active HHV-6 infection has been detected in a variety of cells including CD4+ T cells, monocyte/macrophage cells, dendritic cells, epithelial cells, and astrocytes (Campadelli-Fiume et al., 1999). This means that HHV-6 has a potential of co-infecting KSHV infected cells, especially the cells of monocyte/macrophage lineage and influence KSHV gene expression thorough direct interactions. Indeed, HHV-6 infection has been detected in the monocytes infiltrating KS lesions (Bovenzi et al., 1993; Kempf et al., 1997). It is noteworthy that monocytes support productive KSHV infection and are considered an important reservoir for the maintenance of high KSHV load in KS tumors (Blasig et al., 1997). Furthermore, in an experimental setting where latently KSHV infected cells, BCBL-1 were fused with latently HHV-6 infected JJahan T cells, a reactivation of KSHV from latency was observed, indicating a direct interactions between molecular components of both the viruses (Lu et al., 2005). Although the existence of HHV-6 and KSHV dually infected cells *in vivo* is rare, detection of HHV-6 in a wide range of tissues suggests that HHV-6 can influence KSHV replication thorough soluble factors in a close vicinity of KSHV infected cells (Campadelli-Fiume et al., 1999; Asou et al., 2000). HHV-6 infection may perturb KSHV life cycle by deregulating the expression of a variety of cytokines including interleukin 1β (IL-1β), tumor necrosis factor alpha (TNF-α), and interferons (IFNs) (Flamand et al., 1991; Meeuwsen et al., 2005). Indeed, HHV-6 infection has been experimentally demonstrated to reactivate KSHV lytic replication during co-culture of latently KSHV infected PEL cells with HHV-6 infected T-cells, at least in part through induction of IFN-γ (Lu et al., 2005). Since HHV-6 infection is ubiquitous, reactivation of HHV-6 in the context of immune-suppression may contribute to a progression of KSHV associated pathologies by further augmenting KSHV lytic replication (Lu et al., 2005). A recently published case of systemic co-infection of KSHV and HHV-6 concomitant with very high KSHV viremia in KICS patient support a possible role of HHV-6 in inducing KSHV lytic replication, however, further studies are need to establish HHV-6 infection as a risk-factor for KSHV associated pathologies (Tamburro et al., 2012). Reciprocal role of KSHV in reactivating HHV-6 has not been evaluated yet.

## Co-Infection with Periodontal Pathogens

Periodontal diseases are considered the most common chronic infections in adults (Loesche and Grossman, 2001). They are characterized by chronic inflammation resulting from complex interactions between host-immunity and polymicrobial infections of pathogenic bacteria, viruses, fungi and parasites (Yu et al., 2014). Several herpesviruses including KSHV have been implicated in the occurrence and progression of different forms of periodontal diseases (Ferreira et al., 2011). Immune modulation associated with KSHV infection is likely to favor periodontal pathogenic infection by suppressing host antimicrobial activity, however, specific role of KSHV in the pathogenesis of periodontal disease remains obscure. Conversely, periodontal pathogens have also been implicated in initiation and progression of KS in the oral cavity, a common site for KS lesions (Shiboski and Winkler, 1993). It is estimated that nearly 20% of KS patients first develop KS lesions in the oral cavity, and up to 70% of KS patients eventually develop concurrent oral and cutaneous KS tumors (Pantanowitz et al., 2013; Yu et al., 2014). Oral cavity KS lesions may be seen in all variants of KS but they are more common with AIDS-KS (Fatahzadeh and Schwartz, 2013). A couple of studies have indicated that co-infection of periodontal pathogenic microbiota has a potential to enhance KSHV infectivity and promote lytic replication of the virus (Shiboski and Winkler, 1993; Morris et al., 2007; Dai et al., 2012, 2014; Yu et al., 2014). Secreted metabolic end-products of *Porphyromonas gingivalis* and *Fusobacterium nucleatum*, two of the most common gram-negative oral periodontal pathogens, may induce KSHV lytic replication by increasing the acetylation of histones H3 and/or H4 mediated through the activation of p38 mitogen-activated protein kinase (MAPK) signaling (Morris et al., 2007). Amongst numerous metabolic products released into the oral microenvironment by these two periodontal pathogens, short-chain fatty acids (SCFAs) are the major ones. At least five different SCFAs are present in the saliva of the patients with severe periodontitis, and a majority of these SCFAs act synergistically to promote KSHV lytic replication by increasing histone acetylation, and by decreasing repressive histone trimethylation at the promoter region of the KSHV lytic cascade initiator ORF50/RTA (Yu et al., 2014). Interestingly, SCFAs simultaneously target multiple components of host epigenetic repertoire, which can significantly boost transactivation of KSHV latent genome. SCFAs are well known inhibitors of histone deacetylase class 1 and 2 (HDAC-1 and 2). Additionally, they also down-regulate a class-3 HDAC SIRT1 (silent information regulator-1), and two histone-lysine-*N*-methyl transferases-EZH2 (enhancer of zeste homolog2) and SUV39H1 (suppressor of variegation 3-9 homolog1; Yu et al., 2014). Furthermore, periodontal infection may enhance KSHV infectivity and modulate viral gene expression. Certain pathogen associated molecular patterns (PAMPs) including Lipoteichoic acid (LTA) of a Gram-positive bacteria *Staphylococcus aureus*, and Lipopolysaccharide (LPS) of a Gram-negative bacterium, *P. gingivalis* have been shown to enhance the infectivity of KSHV in nearby oral cells and also influence the expression of viral genes. These PAMPs of oral pathogens may upregulate cellular receptors for KSHV entry, increase production of reactive oxygen species (ROS), and modify intracellular signaling such as MAPK and NF-κB pathway, and consequently affect KSHV entry and/or gene expression (Dai et al., 2014). Collectively, these studies support the notion that development of periodontal disease can be a risk factor for oral Kaposi’s sarcoma. Since periodontal pathogens can make oral microenvironment more conducive for KSHV infection and lytic replication, treatment of oral periodontal disease along with the treatment for Kaposi’s sarcoma may have more favorable outcomes.

## Co-Infection with *Plasmodium Falciparum* and Other Parasites

Malaria is a deadly disease caused by an infection of protozoan parasite *Plasmodium* that is transmitted by the bite of infected female, Anopheles mosquitoes. Currently, five species of *Plasmodium* are known to infect humans of which, *P. falciparum* causes the most severe form of the disease and is accountable for nearly 85% of all malaria cases worldwide. Malaria remains a public health concern accounting for a yearly global death toll of nearly 700,000 people (Schmidt et al., 2015). Interestingly, KSHV infection is highly prevalent in sub-Saharan African countries where malaria is endemic. Based on the high rates of KSHV infection in the regions where malaria is endemic, *Plasmodium* parasitemia has been proposed as a risk factor for KSHV infection and several epidemiological studies have demonstrated that malarial parasite infection is indeed associated with KSHV seropositivity (Wakeham et al., 2011, 2013; Nascimento, 2014; Nalwoga et al., 2015). This speculation is further supported by the fact that the seroprevalence of KSHV and the density of the malarial vector Anopheles mosquitoes in Italy are proportionate (Crispo et al., 2001; Coluzzi et al., 2003; Serraino et al., 2003). Various hypotheses have been proposed to explain how infection with one of these two pathogens could influence the infection by the other pathogen. For instance, malarial parasites are known to induce severe anemia during their blood stages of infection (Haldar and Mohandas, 2009), and severe anemia may trigger KSHV lytic replication by inducing hypoxia, a well characterized condition involved in the reactivation of KSHV from latency and the promotion of KS tumorigenesis (Stolka et al., 2014). As mentioned earlier, a healthy host immune system suppresses KSHV lytic replication, however, impairment of B-cell and T-cell immunity resulting from repeated infections of malarial parasite can lead to viral reactivation and thus could potentially enhance the infectivity and/or transmission of KSHV (Nalwoga et al., 2015). In addition to the immune suppressive effects of malarial parasite infection, Quinine and its derivatives, the drugs that are extensively used to treat malaria, may exert immune suppressive effects on the malaria patient and consequently trigger KSHV lytic replication (Ruocco et al., 2011). Furthermore, person-to-person transmission of KSHV could be facilitated by the bites of female Anopheles mosquitoes along with the transmission of malarial parasites. While experimental evidences demonstrating direct molecular mechanisms of interactions between KSHV and malarial parasite are lacking; Conant et al. (2013) have proposed an interesting potential molecular link between malarial infection and KSHV reactivation. In malaria, the parasite-infected red blood cells (IRBCs) are sequestered from the peripheral circulation by adherence to microvascular endothelium of various organs. This process is critical for the virulence of *P. falciparum*. The sequestration is mediated by direct interactions between the members of *P. falciparum* erythrocyte membrane protein 1 (pfEMP-1), family of parasite encoded proteins that are expressed on the surface of IRBCs and their cognate host receptors expressed on the surface of endothelial cells. CD36 is the main host endothelial cell receptor that mediates the adherence of IRBCs (Gowda et al., 2013). Conant et al. (2013) found that cross-linking of CD36 with a recombinant peptide derived from CD36 binding domain of pfEMP-1 antigen could revoke KSHV latency, presumably through activation of some of the signal transduction pathways that lead to an activation of KSHV-RTA promoter.

Co-infection with other parasites such as helminth and *Mansonella perstans* may influence KSHV pathogenesis by modulating innate and adaptive antiviral host immune response that may result into an enhanced lytic reactivation and/or KSHV infectivity (Damania and Dittmer, 2014). While specific contribution of parasite co-infections on KSHV pathogenesis has not been extensively studied, one cross-sectional study has demonstrated a positive correlation between KSHV seropositivity and parasite co-infection with hookworms or *M. perstans* (Wakeham et al., 2011).

Summing up, malarial and other parasite infections are a likely risk factor for KSHV infection and/or reactivation, but further epidemiological and in-lab studies are needed to underpin the role of parasite co-infections in initiation and progression of KSHV pathogenesis.

## Summary

**FIGURE 1:**
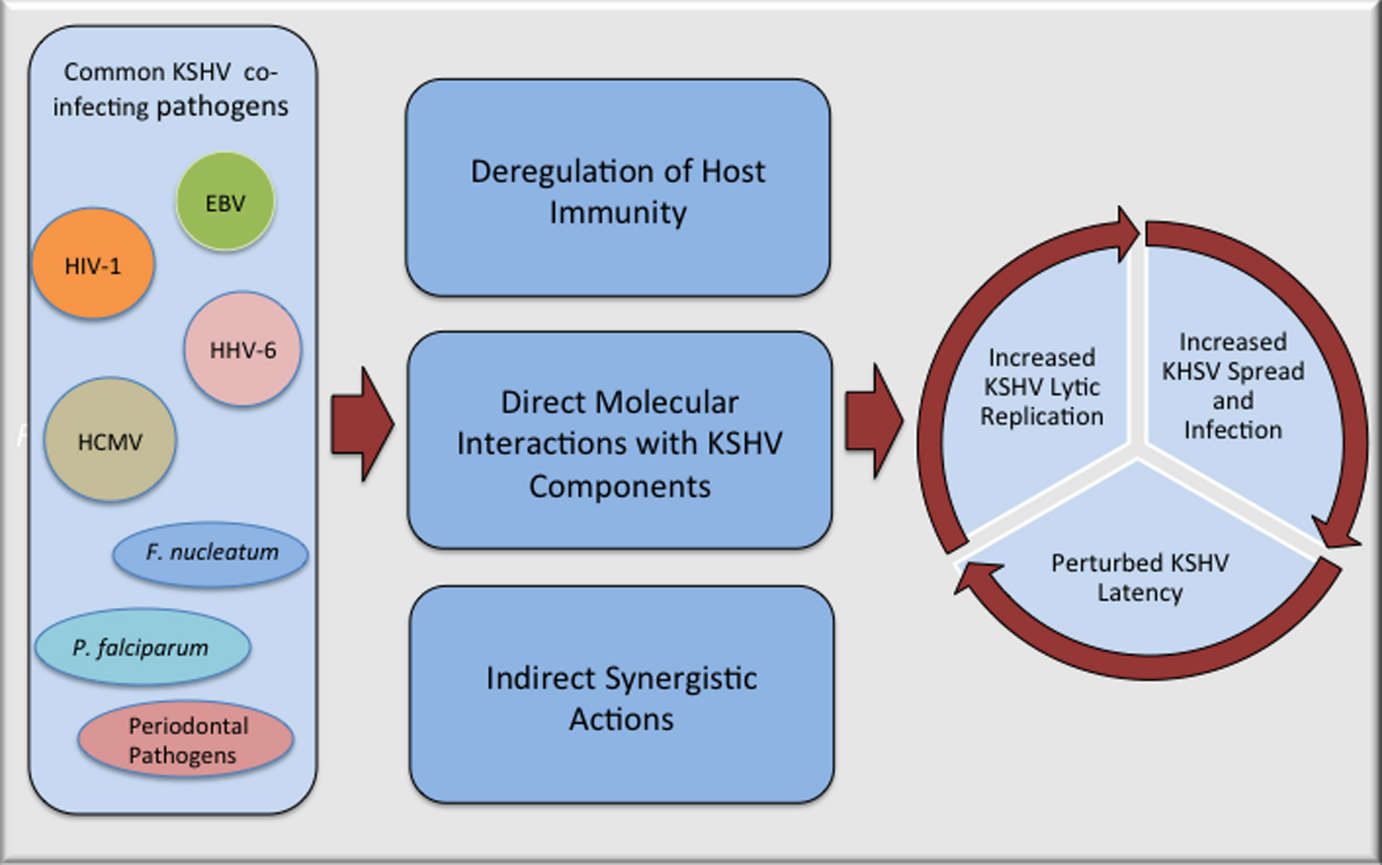
**Potential mechanisms of interactions between Kaposi’s sarcoma-associated herpesvirus (KSHV) and co-infecting pathogens that can accelerate pathogenesis of KSHV associated malignancies.** Many of the commonly observed co-pathogenic infections can perturb KSHV life cycle either through direct physical interactions with KSHV components or in more complex ways that includes suppression of host immunity and manipulation of cellular pathways that can make cellular microenvironment more favorable for KSHV replication and/or spread.

Kaposi’s sarcoma-associated herpesvirus induced pathologies are multi-factorial diseases emerging from extremely complex interactions involving KSHV, countless host factors, and polymicrobial co-infections. While enormously complex interplay of multiple microbial interactions and how co-infected host responds to these interactions with regards to KSHV associated cancers is not well understood, it is clear that co-pathogenic infections have a potential to affect KSHV infection and pathogenesis (**Figure 1**). Additional epidemiological and experimental studies regarding interactions between KSHV and its frequently co-infecting microbes can impart better knowledge of underlying microbial synergy and help in defining new strategies for superior management of KSHV induced pathologies. It is becoming increasingly important to detect co-infecting pathogens and consider synergistic polymicrobial interactions while treating patients for a particular disease, as co-infecting pathogens can impact the overall outcome of the associated pathogenesis through a variety of mechanisms.

## Author Contributions

ST and SV wrote this review.

## Conflict of Interest Statement

The authors declare that the research was conducted in the absence of any commercial or financial relationships that could be construed as a potential conflict of interest.
